# Rest, Reactivity, and Recovery: A Psychophysiological Assessment of Borderline Personality Disorder

**DOI:** 10.3389/fpsyt.2018.00505

**Published:** 2018-10-16

**Authors:** David Eddie, Marsha E. Bates, Evgeny G. Vaschillo, Paul M. Lehrer, Michelle Retkwa, Michael Miuccio

**Affiliations:** ^1^Massachusetts General Hospital, Harvard Medical School, Boston, MA, United States; ^2^Rutgers University – New Brunswick, Piscataway, NJ, United States; ^3^Rutgers Robert Wood Johnson Medical School, Piscataway, NJ, United States

**Keywords:** borderline personality disorder, psychophysiology, central autonomic network, autonomic nervous system, emotion responding, emotion regulation, symptom severity, heart rate variability

## Abstract

Difficulty regulating emotion is a cardinal feature of borderline personality disorder (BPD), yet little is known about the automatic psychophysiological processes involved in this phenotype. Inconsistent findings have emerged from studies that employed limited assessments (e.g., heart rate variability, skin conductance) of autonomic nervous system response to emotional contexts, and compared groups based on the presence or absence of BPD as a categorical diagnosis. This exploratory study assessed a comprehensive set of autonomic nervous system processes in 44 individuals (22 with BPD) at rest, in response to emotionally evocative stimuli, and during a subsequent recovery period. BPD was characterized with a dimensional measure of BPD symptom severity, as a well by categorical diagnosis. At baseline and across experimental tasks, higher heart rate was observed in those diagnosed with BPD compared to controls, and in those expressing greater BPD symptom severity. These effects, however, were fully mediated by differences in physical exercise. In contrast, during recovery from emotional activation, greater symptom severity predicted consistently higher levels of multiple sympathetic and parasympathetic processes compared to lower symptom severity. Overall, these findings suggest that the heart rate elevations sometimes observed in those diagnosed with BPD may be associated with individual and group differences in levels of physical exercise. Results further indicate that adaptive psychophysiological recovery responses following emotional challenge may be disrupted in proportion to BPD symptom severity, independently of exercise. Results highlight the utility of considering lifestyle factors and symptom severity in studies of emotional activation and regulation processes in BPD.

## Introduction

Borderline personality disorder (BPD) is characterized by intense and rapidly shifting emotional states, and difficulty regulating emotion (1–3). While substantial progress has been made in understanding the cognitive components of emotion dysregulation in BPD [or review see (4)], its physiological components remain poorly understood. This knowledge gap limits progress in BPD treatment development because dynamic physiological processes may support or hinder the emotion sensitivity, heightened negative affect, and regulation strategies that have been hypothesized to subserve emotion dysregulation in BPD (4–8). Better understanding of psychophysiological reactivity and regulation processes in BPD therefore may be valuable in uncovering collateral treatment targets in this psychiatric population. That is, although these processes tend occur in an automatic fashion outside of conscious awareness, they are modifiable through empirically supported behavioral techniques [e.g., (9)].

Central control of psychophysiological processes via the autonomic nervous system has been implicated in the ability to adaptively self-regulate emotional responses to changing internal and environmental demands (10, 11). This control is accomplished by the central autonomic network, a brain system that integrates neural signaling in cerebral, limbic, and brain stem areas to modulate physiological activity in coordination with cognitive and emotional demands (5, 12–15). This coordination allows an individual to respond in appropriate magnitude to interoceptive and exteroceptive cues and challenges, and to recover quickly from perturbation (10, 15). It has been posited that individuals with BPD, compared to controls, may be more autonomically activated at rest [e.g., elevated baseline sympathetic activation and/or lower parasympathetic tone; (16–20)], differentially reactive to perturbation [e.g., greater amygdala activation, and paradoxical response to perturbation; (21–24)], and experience more pronounced and sustained negative emotional states during recovery from perturbation [e.g., (25–27)]. Impaired central autonomic network capacity to generate and alter appropriate autonomic responses in synchrony with emotional and other life challenges may lead to maladaptive coping behaviors that further exacerbate, and ultimately maintain, emotion dysregulation in BPD (28–34).

The contribution of impairment of central autonomic network control in disorders of emotion regulation has been studied using proxy measures of heart rate variability and other psychophysiological processes (8). In previous studies of BPD, inconsistent differences have been observed between BPD-diagnosed and control groups in resting state and reactive physiological responses to various emotional stimuli assessed via heart rate (HR) (20, 23, 24, 35, 36), heart rate variability (HRV) (16, 17, 19, 20, 36–40), blood pressure (BP) (41), and skin conductance (SC) (19, 24, 37, 38, 42, 43). Both higher and lower levels of reactivity have been found in BPD compared to healthy controls in response to emotional stimuli (44). When considered together, past results are consistent with a general hypothesis of autonomic dysregulation in BPD, yet a coherent pattern of emotion-related psychophysiological response has not emerged in studies that characterized BPD using a categorical diagnosis.

The present investigation sought to explore some useful next steps to inform future research aimed at better understanding the automatic psychophysiological processes engaged when individuals with BPD experience emotion activation, by simultaneously assessing a comprehensive set of psychophysiological processes at rest, during emotion perturbation, and then during recovery. We utilized multiple psychophysiological indices because any one index, in and of itself, is an incomplete indicator of the operation of a complex psychophysiological regulation system that includes central autonomic control, and the continual feedback between the brain and physiological systems (5).

One potentially important, yet unaddressed consideration that may contribute to the variable results across studies is that the underlying relationships between psychophysiological responding and core elements of psychiatric disorders might be obscured by categorical diagnoses (45, 46). This could be especially relevant to BPD which has a complex, polythetic presentation, with notable individual differences in BPD symptom severity among individuals who meet diagnostic criteria for BPD, as well as among individuals in the general population, albeit at sub-diagnostic levels (47, 48). Compelling arguments have been made for the assessment and study of BPD as a continuous dimension [e.g., (49–53)]; these arguments align with National Institute of Mental Health's Research Domain Criteria, which call for dimensional assessment of mental disorders in research (45, 54). Thus, this study examined the relation of a continuous dimension of BPD symptom severity to autonomic activation, in addition to the presence or absence of a categorical BPD diagnosis.

We considered several other potential contributors to the different results that have been observed: the nature of the emotional stimuli presented, participant exercise behaviors, and the specific psychophysiological processes to be measured.

First, the problem of reliably evoking emotional responses in individuals with BPD is well known (55, 56). Some types of challenges or stressors may differentiate BPD better than others, and differences in findings between previous studies of this population may be related to differences in the stimuli used. A stimulus set that taps into multiple BPD sensitivities may be particularly useful in cue reactivity paradigms in individuals with BPD (55). To this end, a BPD-targeted stimulus set derived by Sloan et al. (56) from the International Affective Picture System (57) was used to engage multiple BPD sensitivities.

Second, although the lifestyle variable of physical exercise is known to markedly affect autonomic nervous system regulation (58, 59), previous studies have not considered potential differences in exercise behaviors between individuals with BPD and controls. As such, the potential moderating effects of participant exercise on psychophysiological indicators of emotion activation were explored.

Third, specific psychophysiological processes measured in previous studies have included minimal, and often non-overlapping sets of psychophysiological assessments. Yet, the examination of simultaneous process indicators can provide insight into multiple complementary and antagonistic cardiovascular responses to challenge that would not be evident in individual indicators [e.g., (60)]. The present study thus included a broad assessment of processes that are dynamic psychophysiological components of emotion activation and recovery mediated by the central autonomic network (13, 15, 61). Dynamic changes in HR, HRV, BP, BP variability, and SC were assessed, as well as baroreflex sensitivity and arterial reactivity.

The baroreflex is a bi-directional brain-body feedback arc composed of parallel, closed-loop branches that coordinate cardiovascular processes to control BP through changes in HR (i.e., the HR baroreflex loop), stroke volume (i.e., the stroke volume baroreflex loop), and vascular tone (i.e., the vascular tone baroreflex loop) (62–65). The efficiency of baroreflex regulation has been related to mood and stress (66, 67). The dynamic regulation of arterial tone in response to vascular tone baroreflex demand is necessary for appropriate hemodynamic perfusion of the brain and organs (68). These psychophysiological processes were assessed in three contexts to capture central autonomic activation across multiple states: at resting baseline while engaged in a standardized low cognitive demand task, during exposure to BPD-related emotional picture cues, and during a naturalistic post-perturbation recovery period.

At baseline, participants with BPD were expected to demonstrate greater HR and SC, and lower HRV in comparison to controls (16–20). During emotional challenge, based on previous findings in BPD and non-BPD samples (19, 20, 29, 40, 69, 70), we expected that both groups would demonstrate responses to emotionally evocative images characterized by sympathetic arousal including increases in HR BP, and SC, and parasympathetic withdrawal characterized by reduced HRV compared to baseline. During the recovery period following emotional challenge, continuing higher levels of HR and SC were expected in the BPD group compared to controls, based on the pronounced and sustained negative emotional states that individuals with BPD are thought to experience after perturbation [e.g., (26, 27)].

These hypotheses also were examined dimensionally to determine whether heterogeneity in BPD symptom severity is related to psychophysiological regulation, regardless of participant's diagnostic status. Examination of processes involved in neurocardiac feedback between the brain and body in the service of emotion responding such as baroreflex sensitivity, cardiodynamic measures, and arterial tone (5, 71), were exploratory as, to our knowledge, they have not been studied in relation to BPD.

## Materials and methods

### Participants

Participants meeting DSM-IV-TR (72) diagnostic criteria (*n* = 22) were recruited using flyers posted at three Dialectical Behavior Therapy clinics in the central New Jersey area that called for volunteers for a research study on emotion responding in people with borderline personality disorder. BPD diagnosis was confirmed by the Structured Clinical Interview for DSM-IV-TR–Section II [SCID-II; (73)]. Complete SCID-I and SCID-II diagnostic information was available from one treatment site for 14 BPD participants. To minimize participant burden at the study session, the remaining eight BPD participants self-reported current and previous psychiatric diagnoses, with BPD diagnosis affirmed using the SCID-II screener. BPD group volunteers were instructed not to deviate from their prescribed medication regimes. For those taking daily doses of a psychiatric medication that could potentially affect physiology (e.g., a benzodiazepine), sessions were scheduled to allow at least 4 h of washout time to minimize acute drug effects. For those taking such medications pro re nata (as needed), the session was rescheduled if medication was taken on the day of the study.

Control participants (*n* = 22) were recruited from the same area via flyers calling for volunteers for a study on emotion responding, and were matched on sex and mean age to BPD participants. Potential control group participants were screened and excluded for any psychopathology using the SCID-I and SCID-II screeners and a brief clinical interview.

For both groups, exclusion criteria included serious, self-reported medical and neurological conditions, clinician assessed active psychosis, medications directly affecting the cardiovascular system (e.g., hypertension medicines), and age less than 18 years. Co-occurring psychopathology and psychiatric medication use were allowed in the BPD group to avoid excluding participants with more severe BPD (74). All consented participants completed the single experimental session. Data from one control participant was excluded for failure to accurately follow experimental instructions.

### Emotional picture stimuli

A stimulus set generated by Sloan et al. (56) was used to capture core themes frequently noted in clinical observations [e.g., (75, 76)] and self-report studies of individuals with BPD [e.g., (77, 78)]. This stimulus set was derived from the International Affective Picture System [IAPS; (79)]. IAPS pictures were rated by 19 clinical and clinical research BPD experts on how “self-referential” they would be to an individual with BPD. Images were judged in terms of how much they depicted or implied a situation that a person with BPD would identify as relevant to their experience. Pictures were ranked by level of self-reference. Sloan et al.'s (56) top 36 ranked pictures were selected for use in the present study. However, because eight of these images are similar in that they depict interpersonal violence, and three contain the same actors in different poses, four of these images (image numbers 6530, 6540, 6550, and 6560) were replaced by other highly ranked, self-referential pictures (2053, 2271, 2800, and 9405). See Eddie and Bates (80) for the complete picture stimuli set and descriptive analysis.

### Self-report measures

BPD dimensional loading was assessed as individuals' total scores on the Borderline Symptom List 23 [BSL-23; (81)], which captures BPD symptom severity. The BSL-23 has high internal consistency (alpha = 0.95), and discriminant validity (82).

Past year exercise behaviors were calculated as number of days on which the participant engaged in physical exercise in past year × average length of exercise sessions.

The following self-report instruments with established reliability and validity were used to characterize the sample: (1) The Acceptance and Action Questionnaire II [AAQ-II; (83)] measured acceptance, experiential avoidance, and psychological inflexibility. (2) The Beck Depression Inventory [BDI; (84)] assessed depression symptom severity. 3) The Difficulties in Emotion Regulation Scale [DERS; (47)] measured difficulties such as lack of awareness, clarity, and acceptance of emotional response, and coping skills repertoire. (4) The Dissociative Experiences Scale II [DES-II; (85)] assessed the frequency of dissociative experiences, which may affect stimulus reactivity (38). (5) Sleep and exercise patterns (86) were assessed as potential covariates of interest that may affect psychophysiology (87). (6) The Positive and Negative Affect Schedule [PANAS; (48)] measured intensity of affect at the present moment (e.g., attentive, enthusiastic, distressed, hostile). (7) The State-trait Anxiety Inventory, Form Y [STAI-Y; (88)] assessed both state and trait anxiety.

## Procedure

Procedures were approved by the University Institutional Review Board for the Protection of Human Subjects. All participants provided written, informed consent. Once informed consent was obtained, control group volunteers completed the SCID-II screener to screen for personality disorder pathology. Relevant sections of the SCID-II interview were administered if necessary to rule out personality disorders. BPD group volunteers for whom full diagnostic history was not available also completed the SCID-II screener. Volunteers found to be ineligible for any reason were compensated at a prorated amount of $10 per hour.

All eligible participants completed the approximate 45-min psychosocial questionnaire battery using Qualtrics survey software. Participants then were seated in a comfortable chair located 2.5 m in front of a large computer screen in a sound attenuated, dimly lit room. Electrocardiograph (ECG) electrodes were placed laterally below the deltoid muscles on the right (–) and left (ground) arms, and in a lateral position above the left ankle (+). A respiration belt was placed across the chest to assess thoracic breathing. Continuous blood pressure was measured using a cuff attached to the middle right hand finger. Electrodermal activity was measured using two stainless steel electrodes affixed to the thenar eminence and hypothenar eminence of the palm of the right hand.

To measure basal physiology, participants first performed a low-demand “vanilla” task (89) for 6 min, in which they viewed colored rectangles on a computer screen while silently counting the number of blue rectangles. Next, participants viewed 36 pictures from the BPD IAPS picture set for a total of 6 min. They viewed each image for 5 s (order randomized), followed by 5 s of black screen during which they verbally reported their subjective arousal using the Self-assessment Manikin, a standardized 9-point Likert scale, [1 = lowest arousal and 9 = greatest arousal; (90)]. Immediately following this task, a 6-min post-challenge recording period ensued where they were asked to sit quietly. No specific instructions were given during this task in order to approximate a “real life” post-challenge recovery period. The entire physiological recording procedure took about 30 min. Participants were compensated $20.

### Equipment and analysis of physiological recordings

ECG, beat-to-beat blood pressure, SC, and respiration were recorded at a rate of 2,000 Hz using a Powerlab Acquisition system (ADInstruments, Colorado Springs, CO) and a Finometer MIDI (Finapres, Amsterdam). Respiration sensors were calibrated pre-session using an 800 ml calibration bag to calculate tidal volume. Physiological data were analyzed using WinCPRS software (Absolute Aliens Oy, Turku, Finland) to transform raw beat-to-beat data into readable waveforms through cubic interpolation and 4 Hz re-sampling.

### Cardiac parameters

Heart beat-to-beat intervals were measured as R-spike to R-spike intervals (RRI) in the ECG waveform. Beat-to-beat stroke volume was calculated from finger pulse recorded by the Finometer MIDI using the Modelflow methodology (91). Cardiac output—the volume of blood pumped by the heart per minute—was calculated as HR × stroke volume. Interbeat interval artifacts were manually identified using WinCPRS software, and manually detrended.

Heart rate variability (HRV), the variation in RRI, was evaluated using time and frequency domain indices. Time domain measurements included the standard deviation of all normal-to-normal intervals (SDNN), the root of the mean squared differences of successive normal-to-normal intervals (RMSSD), and the percent of the number of pairs of adjacent normal-to-normal intervals differing by more than 50 ms (pNN50). In the frequency domain, high frequency (HF: 0.15–0.4 Hz), and low frequency (LF: 0.04–0.15 Hz) HRV indices (61, 92) were calculated using power spectral density analysis (93, 94).

### Vascular parameters

Beat-to-beat peripheral resistance and vascular compliance were calculated using WinCPRS software employing the Modelflow methodology (91) from finger pulse data recorded by the Finometer MIDI. Beat-to-beat pulse transit time was calculated to estimate vascular tone (95, 96), and was measured as time between heart beats (R-spikes of ECG) and the apex of corresponding finger pulse recorded by the Finometer MIDI. Higher pulse transit time corresponds to lower vascular tone.

### Cardiovascular parameters

Beat-to-beat systolic and diastolic blood pressure (SAP and DAP) were measured as the peak and valley, respectively, of each finger pulse recorded by the Finometer MIDI. Mean arterial pressure (MAP) was calculated as [SAP + (2 × DAP)] ÷ 3. Additionally, rate pressure product was calculated as mean HR × mean SAP.

### Baroreflex parameters

Heart rate, stroke volume, and vascular tone baroreflex sensitivities were calculated using cross-spectral analysis through transfer functions (91). Transfer functions were calculated with SAP as the input and RRI, stroke volume, or pulse transit time as the output (91, 93). Sensitivity was estimated as the average power of the transfer function in the low frequency range where coherence between SAP and RRI, stroke volume, or pulse transit time was > 0.5. Heart rate, stroke volume, and vascular tone baroreflex sensitivities reflect the magnitude of change in RRI, stroke volume, and pulse transit time to a one mmHg change in SAP (64, 93, 96).

### Skin conductance

Skin conductance measures included tonic skin conductance level (SCL), and skin conductance reactivity (SCR) (97), where SCR was derived from SCL by filtering out low frequency (0.01–15 Hz) changes that reflect slow responses to ambient temperature (98).

### Respiration parameters

Respiration frequency (breaths per minute), and tidal volume were calculated from the thoracic respiration record for each task (90).

## Statistical analysis

Mixed models were used to examine the effects of BPD diagnosis, experimental task (baseline, stimulus exposure, recovery period), and their interaction on each physiological index. Akaike's information criterion (AIC) and Bayesian information criterion (BIC) scores were used to guide selection of model variance-covariance matrix structure. Unstructured variance-covariance matrices were chosen for all models. In line with the present focus on identifying potential directions for future study, a highly conservative Bonferroni correction was not imposed, rather the model level alpha was set at *p* < 0.001 to reduce the likelihood of type I error. Significant main effects of task were probed using least square means difference tests to assess change in physiology from baseline to stimulus exposure, and stimulus exposure to recovery. Least square means difference tests were used to probe significant group × task interactions.

These models were replicated with BPD symptom severity replacing BPD diagnosis. Significant main effects and BPD symptom severity × task interactions were examined using reduced models, which replicated omnibus models, but with the omission of the recovery period task to test for moderation of psychophysiological changes from baseline to stimulus reactivity by BPD severity, and omission of the baseline task to test for moderation of changes from stimulus reactivity to the recovery period by BPD severity.

## Results

### Sample characteristics

Diagnostic groups did not differ significantly by sex, age, race, body mass index, average hours of sleep per night, and positive affect (*p* > 0.05; Table 1). Participants with BPD, compared to healthy controls, reported more BPD symptoms, state and trait anxiety, depression, present moment negative affect, emotion dysregulation, dissociative symptomology, and less exercise in the past year (Table 1). The BPD group had higher rates of lifetime alcohol, χ^2^(1, *N* = 44) = 8.3, *p* = 0.004 and other drug dependence χ^2^(1, *N* = 44) = 5.6, *p* = 0.003, although groups were similar in terms of past month and past year drinking behaviors (all *p* > 0.05), and other drug use behaviors (all *p* > 0.05).

**Table 1:** Means, standard deviations, and between group differences in participant characteristics and psychological measures by BPD diagnosis group.

	**BPD group mean/freq. *n* = 22**	**Control group mean/freq. *n* = 22**	***t*/χ^2^**	***d***
Sex (number female)	18	18	0.00	0.00
Age	27.9 (8.1)	27.1 (7.7)	0.33	0.10
Body mass index	24.4 (5.9)	23.0 (4.3)	0.87	0.27
**RACE**				
Asian American	2	5		
European American	16	16		
Other or mixed race	4	1		
Percent married	18.2	22.7		
Exercise (days per year × average minutes exercised)	3652.5 (4587.5)	8623.6 (8180.5)	−2.49^*^	0.77
Average hours sleep per night	6.8 (1.6)	7.0 (1.1)	0.44	0.14
Months DBT received lifetime	3.5 (3.5)	0.0 (0.0)	–	–
Months therapy lifetime	70.6 (77.4)	2.6 (6.7)	4.10^*^	1.24
BPD symptoms	40.3 (21.8)	6.8 (8.8)	6.66^*^	2.53
Emotion regulation difficulties	103.0 (22.1)	58.8 (10.6)	8.46^*^	3.09
Dissociative experiences	55.8 (40.5)	15.7 (10.2)	4.51^*^	1.86
State anxiety	38.5 (7.6)	26.9 (5.9)	5.70^*^	1.76
Trait anxiety	47.1 (6.8)	31.6 (6.3)	7.85^*^	2.42
Depression	24.0 (10.8)	4.1 (4.0)	8.13^*^	2.51
Negative affect	19.4 (9.8)	11.4 (3.2)	3.85^*^	1.52
Positive affect	27.5 (8.2)	27.4 (8.0)	0.04	0.01
Acceptance and action	25.4 (5.7)	29.4 (3.7)	−2.73^*^	0.91

Standard deviations in parentheses; BPD, Borderline personality disorder; DBT, Dialectical Behavior Therapy; d, Cohen's d effect size estimate;

* *p < 0.05; Exercise = Number of days exercise per year × average length of exercise sessions; Borderline personality disorder symptomology = Borderline Symptom List 23 (BSL-23); Emotion regulation difficulties = Difficulties in Emotion Regulation Scale (DERS); Dissociative experiences = Dissociative Experiences Scale II (DES-II); State and trait anxiety = State and trait anxiety subscores on the State-trait Anxiety Inventory, Form Y (STAI-Y); Depression = Beck Depression Inventory (BDI); Negative and positive affect = Negative and positive subscales of the Positive and Negative Affect Schedule (PANAS); Acceptance and action = Acceptance and Action Questionnaire (AAQ)*.

Nineteen of 22 participants with BPD had one or more co-occurring Axis-I psychiatric diagnoses. Five of these participants also had a co-occurring Axis-II personality disorder. No control, and 14 BPD participants reported current use of prescribed psychiatric medications that could potentially affect psychophysiological measures. Checks for the potential influence of comorbidity and medication are reported below.

### Data distribution and outliers

With the exception of HR, pNN50, SAP and MAP, the means and variabilities of the physiological indices were notably skewed or kurtotic, and thus were transformed (logarithm or reflect and logarithm); all variables were normally distributed thereafter. To identify multivariate outliers, Mahalanobis distances (99) were calculated for combined HRV indices, and for combined BP indices. Three outliers (Mahalanobis scores with *p* < 0.001) were detected for BP/vascular measures (one BPD participant during stimulus exposure, and one BPD and one control during recovery). Their data were removed from BP/vascular measure analyses. Heart rate baroreflex sensitivity could not be calculated for two BPD participants because coherence between HR and SAP was < 0.5. Their data were omitted from HR baroreflex sensitivity analyses.

### Subjective arousal to emotionally evocative pictures

Participants with BPD reported significantly more subjective arousal during the stimulus exposure than did controls [for details see (80)]. The relationship between BPD symptom severity and mean subjective arousal was of medium effect size (*r* = 0.27), but not statistically significant (*p* > 0.05).

### Psychophysiological differences at resting baseline

The BPD group exhibited significantly higher mean HR, *F*_(1, 42)_ = 4.03, *p* = 0.04, and SCR variability, *F*_(1, 42)_ = 6.61, *p* = 0.02 at baseline compared to the control group (first columns of Tables 2, 3, respectively), though basal differences in mean HR were no longer significant after controlling for exercise. Trends were observed for the relationships between BPD symptom severity and mean HR, *F*_(1, 42)_ = 3.64, *p* = 0.06, and SCR variability *F*_(1, 42)_ = 3.64, *p* = 0.06.

**Table 2:** Average cardiovascular and skin conductance parameters by BPD diagnosis group at baseline, and during stimulus exposure and recovery periods, and *F* statistics from diagnosis group and BPD symptom severity mixed models.

	**Mean (*****SD*****)**			***F***					
	**Baseline**	**Stimulus exposure**	**Recovery period**	**BPD diagnosis group**	**Task^a^**	**BPD diagnosis group × task**	**BPD symptom severity**	**Task^b^**	**BPD symptom severity × task**
**CARDIAC PARAMETERS**									
Heart rate (bpm)				4.51^*^	5.62^*^	0.91	4.74^*^	1.56	0.36
BPD	73.7 (8.5)	74.6 (8.5)	74.9 (8.2)						
Control	68.2 (9.0)	70.0 (8.2)	69.0 (8.9)						
Stroke volume (ml)^c^				0.08	6.41^*^	0.51	0.00	4.48^*^	0.57
BPD	82.6 (20.0)	85.9 (21.5)	81.8 (19.7)						
Control	81.2 (19.3)	82.9 (19.7)	78.2 (16.9)						
Cardiac output (ml/min)^c^				1.20	8.35^*^	1.67	0.84	3.39^*^	0.62
BPD	6111.8 (1858.9)	6491.4 (2174.1)	6151.0 (1986.5)						
Control	5571.8 (1556.3)	5837.3 (1597.7)	5442.4 (1580.2)						
**VASCULAR PARAMETERS**									
Pulse transit time (ms)^c^				2.22	20.79^*^	0.69	0.00	11.58^*^	0.99
BPD	273.5 (20.3)	267.9 (19.2)	272.5 (20.0)						
Control	282.6 (22.1)	277.5 (19.8)	278.5 (16.5)						
Vascular compliance^c^				1.07	10.72^*^	0.65	2.44	5.88^*^	0.13
BPD	1.39 (0.33)	1.34 (0.32)	1.40 (0.30)						
Control	1.31 (0.37)	1.24 (0.35)	1.23 (0.34)						
Peripheral resistance^c^				0.97	1.17	0.20	0.38	0.66	0.04
BPD	15.1 (5.0)	14.6 (4.4)	15.6 (5.9)						
Control	16.5 (5.2)	16.7 (5.3)	17.3 (6.1)						
**CARDIOVASCULAR PARAMETERS**									
Rate pressure product^c^				1.27	34.41^*^	2.59	1.04	14.81^*^	2.70
BPD	9167.4 (1423.3)	9834.0 (2073.2)	9587.1 (1655.6)						
Control	8670.0 (1119.6)	9504.9 (1242.5)	8953.1 (1313.2)						
Systolic arterial pressure (mmHg)				0.24	31.41^*^	1.08	0.33	13.76^*^	2.45
BPD	125.2 (17.6)	131.5 (20.8)	129.1 (20.5)						
Control	128.1 (15.4)	136.6 (15.6)	130.8 (16.6)						
Mean arterial blood pressure (mmHg)				0.02	35.74^*^	2.44	0.44	19.43^*^	3.52^*^
BPD	84.4 (13.1)	86.7 (12.8)	87.5 (18.5)						
Control	84.4 (11.3)	89.3 (11.7)	85.1 (11.2)						
Heart rate Baroreflex^c^				0.03	2.26	0.60	0.89	0.53	0.32
BPD	12.0 (10.1)	13.5 (8.8)	11.2 (5.8)						
Control	11.9 (5.9)	12.3 (5.8)	11.8 (5.2)						
Stroke volume Baroreflex^c^				0.03	0.67	2.65	1.32	0.71	0.48
BPD	0.62 (0.48)	0.58 (0.33)	0.62 (0.39)						
Control	0.53 (0.27)	0.65 (0.26)	0.54 (0.28)						
Vascular tone Baroreflex^c^				0.03	1.49	0.48	1.10	1.31	0.75
BPD	0.53 (0.28)	0.57 (0.35)	0.59 (0.29)						
Control	0.51 (0.24)	0.58 (0.19)	0.57 (0.20)						
**SKIN CONDUCTANCE PARAMETERS**									
Skin conductance level (μS)^c^				2.87	14.86^*^	0.46	0.02	5.76^*^	0.20
BPD	0.40 (5.30)	3.40 (7.60)	1.88 (7.70)						
Control	2.37 (2.90)	4.71 (3.54)	2.81 (4.18)						
Skin conductance response (μS)^c^				0.83	1.21	1.07	0.01	0.35	0.18
BPD	−0.25 (0.79)	−0.04 (0.56)	−0.26 (1.50)						
Control	−0.02 (0.03)	−0.00 (0.02)	−0.00 (0.02)						
**RESPIRATION**									
Respiration frequency^c^				1.72	27.82^*^	0.43	1.96	7.40^*^	3.19
BPD	0.30 (0.06)	0.33 (0.05)	0.28 (0.05)						
Control	0.28 (0.06)	0.31 (0.05)	0.27 (0.05)						
Respiration volume^c^				1.75	5.15^*^	0.98	0.03	2.87	2.26
BPD	350.4 (292.2)	366.2 (284.2)	434.1 (321.9)						
Control	248.1 (136.6)	278.3 (159.4)	290.5 (169.9)						

*Standard deviations in parentheses. BPD, Borderline personality disorder*.

* p < 05. Main effect group df, 1, 42; main effect BPD symptom severity df, 1, 42; main effect task df, 2, 42; interaction terms df, 2, 42; Task

a is main effect of task for BPD group models, Task

b is main effect of task for symptom severity models;

c *Logarithmically transformed data used for analyses; untransformed means are presented*.

**Table 3:** Cardiovascular and skin conductance variability parameters by BPD diagnosis group at baseline, and during stimulus exposure and recovery periods, and *F* statistics from diagnosis group and BPD symptom severity mixed models.

	**Mean (*****SD*****)**			***F***					
	**Baseline**	**Stimulus exposure**	**Recovery period**	**BPD diagnosis group**	**Task^a^**	**BPD diagnosis group × task**	**BPD symptom severity**	**Task^b^**	**BPD symptom severity × task**
**CARDIAC PARAMETERS**									
SDNN (ms)^c^				0.25	4.21^*^	0.11	0.99	1.16	1.00
BPD	52.3 (26.6)	50.7 (19.9)	55.2 (21.0)						
Control	55.4 (27.8)	55.5 (31.2)	58.2 (26.8)						
RMSSD (ms)^c^				0.20	0.51	2.88	0.71	4.82^*^	6.88^*^
BPD	45.8 (33.3)	44.2 (27.7)	45.4 (25.4)						
Control	48.8 (31.1)	47.2 (37.0)	45.8 (33.5)						
pNN50 (%)				0.70	2.89	2.17	2.11	3.50^*^	4.84^*^
BPD	19.8 (21.6)	17.7 (18.2)	19.9 (19.5)						
Control	25.7 (19.3)	23.4 (18.1)	22.6 (18.0)						
High frequency HRV (ms^∧^2)^c^				0.10	0.29	0.56	0.45	1.55	5.01^*^
BPD	978.0 (1789.5)	696.7 (819.9)	718.9 (736.4)						
Control	858.1 (1431.0)	945.1 (2021.8)	921.0 (1708.6)						
Low frequency HRV (ms^∧^2)^c^				0.00	0.89	1.01	0.26	1.32	1.10
BPD	910.4 (778.8)	925.5 (911.3)	1030.8 (992.7)						
Control	984.3 (1158.0)	1096.6 (1332.0)	875.6 (822.0)						
Stroke volume variability (ml)^c^				0.03	0.33	2.00	0.07	0.27	1.67
BPD	6.09 (3.26)	5.47 (2.43)	6.29 (2.44)						
Control	5.95 (2.37)	6.17 (2.54)	5.54 (2.12)						
Cardiac output variability (ml/min)^c^				1.05	0.45	2.42	0.06	0.32	0.72
BPD	574.5 (326.0)	535.7 (206.9)	606.2 (273.6)						
Control	477.7 (234.4)	540.5 (251.5)	479.5 (219.0)						
**VASCULAR PARAMETERS**									
Pulse transit time variability (ms)^c^				0.51	2.60	2.33	0.19	0.17	4.02^*^
BPD	4.96 (1.57)	4.65 (1.29)	5.43 (1.52)						
Control	5.35 (1.63)	5.38 (2.06)	5.14 (1.46)						
Vascular compliance variability^c^				0.15	0.70	3.89^*^	1.43	1.81	5.39^*^
BPD	0.08 (0.05)	0.07 (0.04)	0.10 (0.06)						
Control	0.08 (0.05)	0.08 (0.04)	0.07 (0.04)						
Peripheral resistance variability^c^				0.26	1.08	2.82	0.30	0.64	3.63^*^
BPD	1.50 (1.09)	1.21 (0.54)	1.70 (1.30)						
Control	1.58 (1.22)	1.71 (1.19)	1.61 (1.15)						
**CARDIOVASCULAR PARAMETERS**									
Rate pressure product variability^c^				0.24	7.07^*^	2.93	0.04	1.32	5.49^*^
BPD	673.4 (282.8)	647.1 (221.3)	772.5 (280.9)						
Control	627.6 (227.6)	663.3 (200.9)	675.4 (245.1)						
Systolic arterial pressure variability (mmHg)				0.00	1.61	1.50	0.31	0.30	1.71
BPD	5.57 (2.05)	5.14 (2.12)	5.95 (2.17)						
Control	5.85 (2.40)	5.54 (2.23)	5.56 (2.36)						
Mean arterial pressure variability (mmHg)				0.07	3.47^*^	1.58	0.39	0.05	3.00
BPD	3.75 (1.19)	3.59 (1.14)	4.30 (1.60)						
Control	3.80 (1.00)	3.78 (0.85)	3.82 (1.18)						
**SKIN CONDUCTANCE PARAMETERS**									
Skin conductance level variability (μS)^c^				1.58	3.88^*^	0.03	1.66	0.95	0.39
BPD	1.88 (1.43)	1.53 (1.21)	1.47 (1.56)						
Control	1.51 (1.48)	1.27 (1.79)	0.97 (1.09)						
Skin conductance response variability (μS)^c^				6.82^*^	1.09	0.90	2.30	3.66^*^	0.89
BPD	0.68 (0.85)	0.75 (0.90)	0.62 (0.84)						
Control	0.21 (0.18)	0.34 (0.35)	0.23 (0.33)						

*Standard deviations in parentheses. BPD, Borderline personality disorder; SDNN, Standard deviation of all normal-to-normal intervals; pNN50, percent of normal-to-normal adjacent intervals greater than 50 ms; RMSSD, Root of the mean squared differences of successive normal-to-normal intervals*.

* p < 0.05. Main effect group df, 1, 42; main effect BPD symptom severity df, 1, 42; main effect task df, 2, 42; interaction terms df, 2, 42; Task

a is main effect of task for BPD group models, Task

b is main effect of task for BPD symptom severity models;

c *Logarithmically transformed data used for analyses; untransformed means are presented*.

## Repeated measures mixed models

The mixed model results are reported in Tables 2, 3. Significant main and interaction effects are reported only when model level alphas were *p* < 0.001.

### Main effects of diagnostic group and BPD symptom severity

There were significant main effects of diagnostic group and BPD symptom severity on HR, wherein participants with BPD and those with greater symptom severity had higher HR across tasks than did controls and participants with lower BPD severity (Table 2). These main effects were no longer significant after the exercise measure was added to models (*p* > 0.05). Exercise significantly predicted HR in the BPD diagnosis model, *F*_(1, 41)_ = 4.72, *p* = 0.04, and in the dimensional model, *F*_(1, 41)_ = 5.67, *p* = 0.04. The only other significant main effect of group was observed for SCR variability. *Post hoc* testing showed participants with BPD demonstrated greater SCR variability across tasks. Including exercise in this model did not significantly alter its results.

### Main effects of task

Both diagnostic group and BPD severity mixed models yielded significant main effects of task on stroke volume, cardiac output, pulse transit time, compliance, rate pressure product, SAP, MAP, and SCL (Table 2). Main effects of task for HR, SDNN, rate pressure product variability, MAP variability, and SCL variability were observed in diagnostic group models. Main effects of task were observed for RMSSD, pNN50, and SCR variability in the BPD severity mixed models (Table 3). Entering exercise in these models did not significantly alter their results.

For the majority of indices, where main effects of task were observed in the diagnostic group models, they were also observed in the BPD dimensional models. For these indices, to avoid repetition, *post hoc* tests are only reported for the diagnostic group models. Additionally, for indices where main effects of task were only observed in the dimensional models (i.e., RMSSD, pNN50, SCR variability), *post hoc* results are reported for these models.

Overall, *post hoc* testing for main effects of task demonstrated expected increases in HR, BP, and SC in response to stimulus exposure. From stimulus exposure to the recovery period, participants demonstrated significant reductions in BP and SC, and increases in SDNN, indicating recovery from arousal.

Specifically, increases from baseline to stimulus exposure were observed in mean HR, *t*_(42)_ = 3.24, *p* = 0.002, mean stroke volume, *t*_(42)_ = 2.26, *p* = 0.03, mean cardiac output, *t*_(42)_ = 3.67, *p* = 0.0007, mean rate pressure product, *t*_(42)_ = 6.98, *p* < 0.0001, mean SAP, *t*_(42)_ = 6.44, *p* < 0.0001, mean MAP, *t*_(42)_ = 4.76, *p* < .0001, mean SCL, *t*_(42)_ = 3.86, *p* = 0.0004, mean respiration frequency, *t*_(42)_ = 5.07, *p* < 0.0001, and in the BPD symptom severity models, SCR variability, *F*_(1, 42)_ = 7.27, *p* = 0.01. Decreases were observed in mean pulse transit time, *t*_(42)_ = −6.38, *p* < 0.0001, mean compliance, *t*_(42)_ = −4.79, *p* < 0.0001, and SCL variability, *t*_(42)_ = −2.30, *p* = 0.03.

From stimulus exposure to stimulus recovery, increases were observed for SDNN, *t*_(42)_ = 2.06, *p* = 0.04, mean pulse transit time, *t*_(42)_ = 3.40, *p* = 0.002, rate pressure product variability, *t*_(42)_ = 2.43, *p* = 0.02, and respiration volume, *t*_(42)_ = 2.19, *p* = 0.04, while decreases were observed for mean stroke volume, *t*_(42)_ = −3.54, *p* = 0.001, mean rate pressure product, *t*_(42)_ = −3.09, *p* = 0.004, mean cardiac output, *t*_(42)_ = −3.02, *p* = 0.004, mean SAP, *t*_(42)_ = −4.08, *p* = 0.0002, mean MAP, *t*_(42)_ = −4.08, *p* = 0.0002, mean SCL, *t*_(42)_ = −3.34, *p* = 0.002, and respiration frequency, *t*_(42)_ = −7.14, *p* < 0.0001.

All other *post hoc* tests for main effects of task were non-significant (*p* > 0.05).

### BPD × task interactions

There were no significant BPD diagnostic group by task interaction effects for HR, HRV, BP, or SC (Tables 2, 3), suggesting that the groups were similar in terms of their response to stimulus exposure and recovery. The only significant interaction effect was observed for vascular compliance variability (Table 3), although *post hoc* tests did not reveal significant changes in this measure from baseline to exposure, or exposure to recovery (*p* > 0.05).

In contrast, the mixed models for BPD symptom severity gave rise to significant interaction effects for MAP (Table 2), the HRV measures of RMSSD, pNN50, and HF HRV, as well as vascular pulse transit time, compliance, peripheral resistance and rate pressure product variability parameters (Table 3). *Post hoc* tests indicated that participants responded similarly to stimulus exposure (i.e., autonomic change from baseline to stimulus exposure; all *p* > 0.05). However, from exposure to recovery, greater BPD severity predicted increases in RMSSD, *F*_(1, 42)_ = 7.16, *p* = 0.01, pNN50, *F*_(1, 42)_ = 9.14, *p* = 0.004, and HF HRV, *F*_(1, 42)_ = 8.24, *p* = 0.007, while lower severity predicted decreases in these measures. Adding exercise to these models did not significantly alter these results. In the exploratory analyses of vascular and cardiovascular variability, greater BPD severity predicted sustained or increased pulse transit time variability, *F*_(1, 42)_ = 7.69, *p* = 0.008, compliance variability, *F*_(1, 42)_ = 5.88, *p* = 0.02, peripheral resistance variability, *F*_(1, 42)_ = 4.97 *p* = 0.03, and rate pressure product variability, *F*_(1, 42)_ = 9.88, *p* = 0.003 from exposure to recovery.

### Checks for effects of respiration, medication, sex, age, and dissociative tendencies

Respiration frequency and respiration volume were not correlated with symptom severity, or any physiological change measure (all *p* > 0.05). Significant main effects of task were observed for respiration frequency and respiration volume models (Table 2).

Student's *t*-tests showed that participants in the BPD group taking medication were not significantly different from those not taking medication on physiological measures at baseline, during stimulus exposure, or during the recovery period (all *p* > 0.05). Point biserial correlation indicated that BPD symptom severity was not related to BPD participants' medication status (yes/no), *p* > 0.05. Adding medication status (yes/no) as covariate to mixed models did not significantly alter results, nor were any main effects of medication observed.

Entering sex in mixed models did not significantly alter their results. A main effect of sex was observed only for compliance variance in the BPD diagnostic group model, *F*_(1, 41)_ = 4.93, *p* = 0.03.

Although the diagnostic groups were matched for age, the potential influence of age in the BPD symptom severity models was checked. Adding age as covariate to the BPD severity mixed models did not significantly alter results, nor were any main effects of age observed (all *p* > 0.05).

Because transient dissociation is sometimes observed in individuals with BPD, total DES-II scores reflecting participants' dissociative tendencies were added as a covariate and all mixed models were re-estimated. Including DES-II scores in these models did not significantly alter results, nor were there any main effects of dissociation.

## Discussion

The present investigation sought to inform future research aimed at better understanding the autonomic nervous system processes contributing to emotion activation and recovery in BPD by comprehensively assessing psychophysiology at rest, during emotional challenge, and during recovery, with special consideration given to the potential influence of BPD symptom severity and physical exercise.

With respect to diagnostic group differences at resting baseline, the BPD group had significantly higher mean HR and SCR variability than did the control group. At face value, these findings provide partial support for the hypothesis of heightened baseline sympathetic nervous system activation in this disorder. The group difference in mean HR, however, was no longer significant after controlling for exercise. Thus, it is possible that previously observed differences in HR between those diagnosed with BPD and controls may have been a function of lifestyle-related behaviors such as exercise engagement, rather than an intrinsic disorder-related difference in resting state arousal. Contrary to hypothesis, there were no baseline differences in HRV indices thought to reflect parasympathetic tone such as pNN50 and HF HRV. Overall, the baseline results suggest a difference in the balance of sympathetic and parasympathetic mediation of HR between BPD participants and controls, which may be due to decreased vagal influence and/or increased sympathetic influence on HR related to less physical activity.

In the mixed model analyses, BPD diagnosis, and greater BPD symptom severity predicted higher mean HR across rest, emotional challenge, and recovery compared to no BPD diagnosis or lower severity, while having a BPD diagnosis was associated with greater SCR variability. Adding exercise to the HR models, however, resulted in the main effects of diagnostic group and symptom severity on mean HR to become non-significant. This indicates heightened HR in BPD across contexts was linked to reduced physical activity levels and suggests that individuals with BPD may particularly benefit from increased exercise to reduce the influence of vagal withdrawal and/or sympathetic activation in response to emotional challenge, and during recovery from challenge, as well as at rest. Although greater SCR variability in participants with BPD was suggestive of greater sympathetic activation, the lack of clear differences between groups on other indices reflecting sympathetic innervation perhaps points to the need for a more nuanced examination of alpha and beta sympathetic systems in BPD, which each drive distinct physiological processes in the body.

The mixed models also produced notable moderation effects wherein BPD symptom severity interacted with task demands in affecting changes in cardiac and vascular variabilities during recovery from emotional challenge. These effects were not significantly influenced by exercise. As hypothesized, higher BPD symptom severity predicted sustained, or increased autonomic activation from stimulus exposure to recovery, as reflected by variability indices mediated in part by the sympathetic nervous system (rate pressure product, pulse transit time, compliance, and peripheral resistance variability). Lower BPD loading, conversely, was associated with reductions in mean rate pressure product and the sympathetically mediated variability indices, suggesting the expected pattern of sympathetic withdrawal following removal of the emotional challenge. This pattern of findings is generally consistent with theories suggesting BPD is characterized by a slow return to emotional baseline after perturbation (26, 33), and further points to greater symptom severity as a key consideration.

BPD symptom severity also positively predicted HRV changes (i.e., RMSSD, pNN50, and HF HRV), such that greater severity predicted increases in HRV from stimulus exposure to recovery. It is possible that this increased parasympathetic activity reflected an immediate compensatory autonomic response to the sustained or increasing sympathetic activation experienced during the recovery period by those with greater BPD symptom severity. Low levels of the BPD symptom severity, on the other hand, were associated with decreased or stable HRV levels from stimulus exposure to stimulus recovery. Thus, the normative sympathetic withdrawal associated with low levels of severity, observed primarily in healthy controls, may have been a sufficient adaptation during recovery that did not evoke or trigger a parasympathetic counter-response. These speculations would be useful to empirically test in future studies that are more highly powered and that examine emotion challenges of varying nature and intensity.

Contrary to prediction, there were no significant main effects of diagnostic group, or diagnosis by task interaction on any other index of psychophysiological regulation, including variability, with one exception (vascular compliance variability). These null findings for the diagnostic group models do not appear to be attributable to lack of task sensitivity. The task manipulation was successful in significantly affecting the mean values of virtually all of the cardiac, vascular, cardiovascular, and SC parameters (Table 2). The effects were generally large indicating that the emotional picture cue exposure and recovery tasks provoked meaningful systems-level autonomic changes in the sample as a whole. All mean indices of cardiac processes showed a consistent pattern of increase from baseline to emotional stimulus exposure, followed by a decrease from exposure to recovery period. These changes were consistent with the intended difference in the relative energy/metabolic demands of the low cognitive-demand rest, emotional challenge, and recovery periods. Participants' vascular processes of pulse transit time and compliance decreased during emotional picture challenge. These results suggest that vascular tone increased during exposure, consistent with a sympathetic nervous system response to increased energy demands, which was also reflected in the direction of the SCL changes that were observed between tasks. Further, there was a significant increase in average pulse transit time during the recovery period, indicative of relaxation in participants' vascular tone when the emotional challenge was removed.

Variability in psychophysiological processes, such as HRV, as well as less studied vascular tone and BP variability that characterize the regulation quality of the cardiovascular functions, also were explored to provide insight into real-time adaptive responding (Table 3). Significant, increases in SDNN, RMSSD, and pNN50 indicative of adaptive reaction of the cardiovascular system to change, were observed from stimulus exposure to the recovery period, consistent with an expected increase in relaxation during recovery. Additionally, variability of rate pressure product, mean arterial pressure, and SCL—processes that closely link to energy/metabolic demands—were affected by task. This pattern of results shows that the baseline, exposure, and recovery task manipulations gave rise to measurable, mean-level cardiovascular system responses that were consistent with the intended design of the study.

The present investigation was the first to study the baroreflex mechanism in BPD. In both the categorical and dimensional models, no significant effects of BPD diagnosis or symptom severity on baroreflex were observed. The lack of evidence for diminished sensitivity in these brain-cardiovascular feedback loops supports the idea that lifestyle modifications such as increased physical activity may be effective in reducing psychophysiological indicators of heightened reactivity in BPD, such as increased HR. At the same time, heightened symptom severity was related to a broad spectrum of aberrant autonomic activity during recovery from challenge that was not related to exercise levels. For example, during recovery, vascular tone and HR variability parameters were more active in the context of high symptom severity. Taken together, results tentatively suggest an allostatic state (31) in BPD that may be primarily due to lifestyle-related elevations in HR, and possibly SCR variability. Superimposed on this allostatic state, psychophysiological variability index changes were observed in those with relatively higher BPD symptom severity that may indicate adaptive, peripheral allostatic accommodation responses after exposure to emotionally challenging picture cues (100). This interpretation is consistent with the likelihood that the magnitude of the load during recovery would increase with the severity of BPD symptoms.

The central autonomic network includes brainstem as well as midbrain and forebrain structures that overlap with core emotion activation and regulation areas of the brain (e.g., brainstem nuclei, amygdala, prefrontal cortex). These brain areas are thought to be the first to show “wear and tear” as a consequence of stress exposure (100) leading not only to brain changes that have been observed in this disorder (101), but also to changes in autonomic function and peripheral physiology (100). The present findings suggest there are automatic cardiovascular processes engaged during recovery from emotional challenge in BPD, and given unimpaired baroreceptor sensitivity, these peripheral systems can still mount an adaptive response. At the same time, these findings point to potential ways in which adaptive autonomic nervous system responses to stressors may—in the context of heightened BPD severity—increase allostatic load over time and compromise this system [e.g., (31, 102)]. Further study is needed to determine if continued allostatic load changes set points in such a way as to ultimately undermine adaptive psychophysiological contributions to emotional response (102).

**Figure 1:**
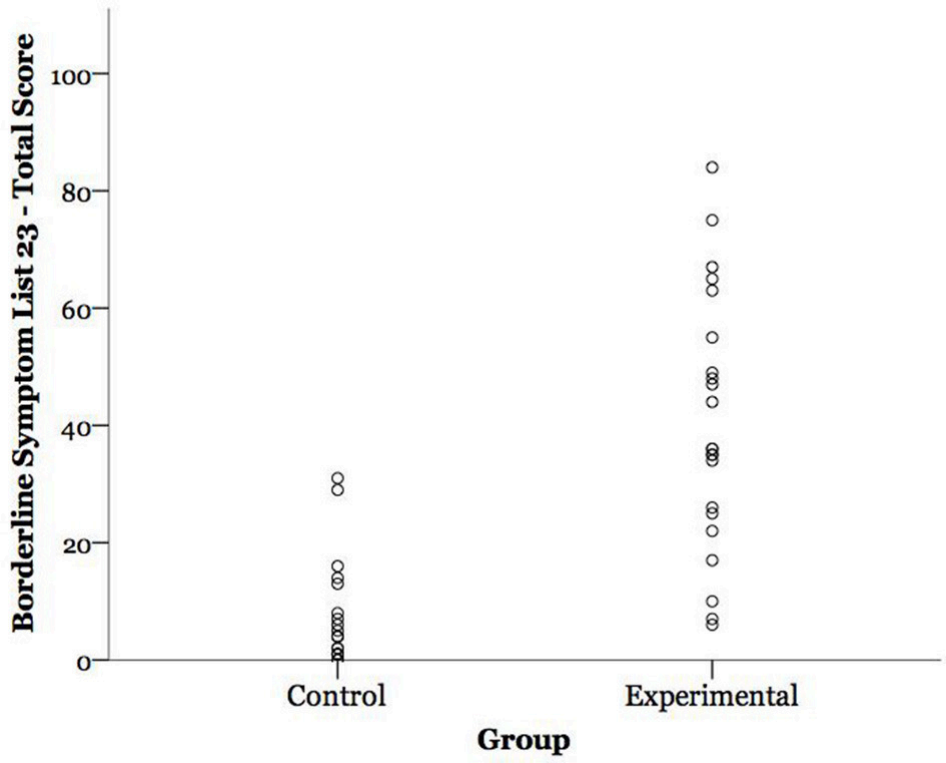
Participants' borderline personality disorder (BPD) symptom severity scores by group. BPD symptom severity is expressed as participants' total score on the Borderline Symptom List 23 (BSL-23); possible range = 0–92.

The present findings highlight the importance of considering BPD symptom severity when studying this disorder, in that the range of BPD symptom severity in different BPD samples may obfuscate diagnostic-control differences (see Figure 1). Symptom severity has been a useful dimensional approach to better understand the pathophysiology of other psychiatric disorders such as depression, anxiety, and post-traumatic stress disorder (103), conditions that often co-occur with BPD along with subclinical presentations of depression (104). We note that BPD symptom severity was highly correlated in this sample with emotion dysregulation (DERS, *r* = 0.88, *p* < 0.0001), a core feature of BPD. This observation speaks to the potential explanatory value of studying dimensional features of BPD, rather than sole use of categorical diagnoses. These findings suggest future research will benefit from assessing severity of BPD presentation, and that divergent findings in the BPD psychophysiology literature may be in part explained by a failure to consider heterogeneity in this disorder. More broadly, the present findings highlight the importance of considering symptom severity and lifestyle factors when studying the psychophysiological corollaries of psychiatric disorders, given that autonomic processes, like psychiatric disorders, are dynamic and lie on a continuum.

### Limitations and future directions

Several limitations in the present investigation bear consideration. The modest sample size limited the study's power to identify statistically significant effects, and replication with larger samples is needed to increase confidence in the pattern of effects found in this study. While the IAPS images used in this study to evoke an emotional response were selected by expert consensus to characterize a range of BPD sensitivities, and participants with BPD in this study reported significantly higher subjective arousal to the pictures than controls, we previously reported that there was heterogeneity across different picture cues in how arousing the present sample rated the stimulus set (80). More work is needed to build a more consistently challenging stimulus set to increase the potency of the emotionally arousing visual stimuli in future BPD studies. It also should be noted that while allowing for psychiatric medications within the BPD group reduced the risk of excluding participants with more severe BPD, it is possible that medication may have affected results, even though including a medication covariate in the mixed models did not significantly alter results. Similarly, allowing for comorbidity in the BPD group reduced the likelihood of excluding individuals with more severe BPD, who are more likely to meet criteria for other psychological disorders, yet also may have affected results in unpredictable ways; though the BPD symptom severity measure may have captured some of the between group variance in negative affect, it is possible the present findings are not wholly specific to BPD. Finally, all participants were recruited from clinics providing Dialectical Behavior Therapy, which may potentially reduce the generalizability of the present results. Future studies are needed to replicate and extend these results in larger samples so that potential effects of exercise, medication, and psychiatric comorbidity can be parsed out. It is possible that lifestyle changes, such as increased exercise, warrant further attention in individuals with BPD.

## Author contributions

DE and MB designed the study with EV and PL providing design suggestions. DE, EV, MR, and MM were responsible for data collection and preparation. DE, MR, and MM conducted literature reviews for the manuscript; DE conducted all analyses; DE and MB wrote the manuscript with EV and PL contributing edits.

### Conflict of interest statement

The authors declare that the research was conducted in the absence of any commercial or financial relationships that could be construed as a potential conflict of interest.
